# Endothelial cell division in angiogenic sprouts of differing cellular architecture

**DOI:** 10.1242/bio.012740

**Published:** 2015-09-14

**Authors:** Vahap Aydogan, Anna Lenard, Alexandru Stefan Denes, Loic Sauteur, Heinz-Georg Belting, Markus Affolter

**Affiliations:** Biozentrum der Universität Basel, Klingelbergstrasse 70, Basel CH-4056, Switzerland

**Keywords:** Junctional dynamics, Cytokinesis, Endothelial cell division, Multicellular tube, Unicellular tube, Lumen, Live-cell imaging, Actin distribution

## Abstract

The vasculature of the zebrafish trunk is composed of tubes with different cellular architectures. Unicellular tubes form their lumen through membrane invagination and transcellular cell hollowing, whereas multicellular vessels become lumenized through a chord hollowing process. Endothelial cell proliferation is essential for the subsequent growth and maturation of the blood vessels. However, how cell division, lumen formation and cell rearrangement are coordinated during angiogenic sprouting has so far not been investigated at detailed cellular level. Reasoning that different tubular architectures may impose discrete mechanistic constraints on endothelial cell division, we analyzed and compared the sequential steps of cell division, namely mitotic rounding, cytokinesis, actin re-distribution and adherence junction formation, in different blood vessels. In particular, we characterized the interplay between cell rearrangement, mitosis and lumen dynamics within unicellular and multicellular tubes. The lumen of unicellular tubes becomes constricted and is ultimately displaced from the plane of cell division, where a *de novo* junction forms through the recruitment of junctional proteins at the site of abscission. By contrast, the new junctions separating the daughter cells within multicellular tubes form through the alteration of pre-existing junctions, and the lumen is retained throughout mitosis. We also describe variations in the progression of cytokinesis: while membrane furrowing between daughter cells is symmetric in unicellular tubes, we found that it is asymmetric in those multicellular tubes that contained a taut intercellular junction close to the plane of division. Our findings illustrate that during the course of normal development, the cell division machinery can accommodate multiple tube architectures, thereby avoiding disruptions to the vascular network.

## INTRODUCTION

Embryonic organogenesis requires nutrient delivery, gas exchange and metabolic waste removal, and these processes depend upon the presence of a functional cardiovascular system. Therefore, the vascular network assembles early during development from endothelial cells (ECs) and grows into a dynamic network of hollow tubes that directs blood flow to the developing organs. Most of the later born blood vessels are the product of angiogenesis, a process in which new branches sprout from pre-existing blood vessels. During development, the vascular network becomes more elaborate through the opening of new circulatory pathways, a process known as anastomosis (Adams and Alitalo, 2007; Wacker and Gerhardt, 2011). As we have recently shown (Herwig et al., 2011; Lenard et al., 2013) anastomosis entails a stereotypic set of cell behaviors and can occur either between two sprouts that contact each other, or through the fusion of a sprout with a pre-existing, perfused blood vessel.

The vasculature of the zebrafish trunk consists of segmental arteries (SAs) that branch out of the dorsal aorta (Isogai et al., 2001). A vascular sprout contains several stalk cells and a leading tip cell (Blum et al., 2008; Siekmann and Lawson, 2007). SAs are arranged in a metameric pattern and their tip cells eventually contact each other, leading to vessel anastomosis and the formation of the dorsal longitudinal anastomotic vessel (DLAV) (Herwig et al., 2011; Lawson and Weinstein, 2002). During the formation of the trunk vasculature, approximately from 20 and 50 hpf, the endothelial cells of the SAs and the DLAV undergo extensive proliferation (Blum et al., 2008; Lawson and Weinstein, 2002). Therefore, the interplay of the cellular mechanisms of junctional remodeling, lumen formation and EC division can be investigated through live imaging during this time window (Ellertsdottir et al., 2010; Lawson and Weinstein, 2002). It has been shown that the ECs within the SAs can reorganize their intercellular junctions and apical domains, through a cell-autonomous process that does not require blood pressure (Herwig et al., 2011). Neighboring cells were observed to crawl over each other, expanding their shared subapical contacts and their apical surface until the vascular lumen was stabilized by multiple cells (Blum et al., 2008; Herwig et al., 2011; Lenard et al., 2013; Phng et al., 2015).

In terms of morphology and cellular plasticity, SA cells fall somewhere in-between migrating cells (at the tip) and cells that are tightly built into epithelial sheets (in the stalk). Tip cells are characterized by abundant filopodia at the leading edge, befitting their migratory nature and are followed by the trailing stalk cell (Gerhardt and Betsholtz, 2003; Siekmann and Lawson, 2007). By contrast, stalk cells undergo an extension of their cell surface and junctional contacts and are mostly arranged into multicellular tubes (Herwig et al., 2011; Sauteur et al., 2014).

Cell division has been extensively investigated using *in vitro* culture, and the cellular and molecular mechanisms of the mitotic machinery are well understood. The first step is mitotic rounding, a generic feature of cell division that is driven by changes in the shape and the rigidity of the cell cortex (Cadart et al., 2014). It has been shown that this actomyosin-driven process is necessary for the proper assembly, maintenance and orientation of the central spindle (Kunda et al., 2008; Lancaster et al., 2013; Rosenblatt et al., 2004). Spindle orientation subsequently defines the plane of cell division through the accumulation of phosphorylated Myosin II at the plasma membrane, which drives the assembly of a contractile ring (reviewed in Fededa and Gerlich, 2012; Green et al., 2012; Levayer and Lecuit, 2012). The next step is the partitioning into two daughter cells, or cytokinesis, which takes place shortly after chromosome segregation. During cytokinesis, the actomyosin ring contracts and eventually collapses to a small intercellular bridge, the so called midbody (Green et al., 2012). Finally, the severing of the constricted plasma membrane, a process known as abscission, marks the end of mitosis.

Within epithelial sheets or tubes, dividing cells maintain the adherens junctions (AJs), which confer tissue integrity (Bourdages and Maddox, 2013; Nakajima et al., 2013). However AJs are extensively reconstructed during mitotic rounding and cytokinesis (Harris and Tepass, 2010; Herszterg et al., 2014). The neighboring ECs exert forces on the mitotic cell through cadherin proteins (the core of AJs) that are, in turn, linked to the actomyosin cortex (Harris and Tepass, 2010). Morphogenetic movements such as cell intercalation and invagination require a degree of synchronization between junctional re-arrangement and mitosis (Kondo and Hayashi, 2013; Levayer and Lecuit, 2012).

Because of their three-dimensional structure, tubular networks have a more complex morphology than epithelial sheets. Therefore, the division of elongated and lumenized cells may require some adaptations of the mitotic machinery in order to accommodate their peculiar geometry as was recently shown in a study of the *Drosophila* larval trachea system (Denes et al., 2015). While the actomyosin rings that drive cytokinesis in the *Drosophila* epithelia are able to symmetrically deform the AJs of the two cells that flank the emerging junction (Founounou et al., 2013; Guillot and Lecuit, 2013; Herszterg et al., 2013), during cytokinesis in tracheal tubes, the membrane furrows asymmetrically on the side of the cell that is proximal to the nucleus, and the new junction then extends around the lumen until it connects and fuses with another membrane. We found that in the remodeling dorsal tracheal branches, such asymmetric *de novo* junction formation is the norm, presumably because the specific geometry and the rigidity of the tubes favor this outcome (Denes et al., 2015).

The integration of proliferative and morphogenetic processes is therefore critical for proper vessel morphogenesis (Zeng et al., 2007). However, it has not been investigated in detail how EC division proceeds in a dynamic environment, in which lumen formation and cell rearrangements occur concomitantly and vessel integrity has to be maintained. Here, we investigated the interplay between cell division, junctional rearrangement, actin distribution and lumen dynamics during SA morphogenesis in the zebrafish, using an array of fluorescently labeled markers and confocal live imaging. We find that membrane furrowing during cytokinesis is symmetric in unicellular tubes and in those multicellular tubes with a cylindrical symmetry. ECs in a multicellular DLAV may undergo either symmetric or asymmetric cytokinesis, depending on the orientation of the intercellular junctions relative to the plane of division. We also found that, unlike the chitin-reinforced lumen of *Drosophila* trachea (Denes et al., 2015), the flexible lumen of unicellular vascular tubes can collapse during mitotic rounding and cytokinesis.

## RESULTS

### The cellular organization of SA branches and of the DLAV

**Fig. 1.**
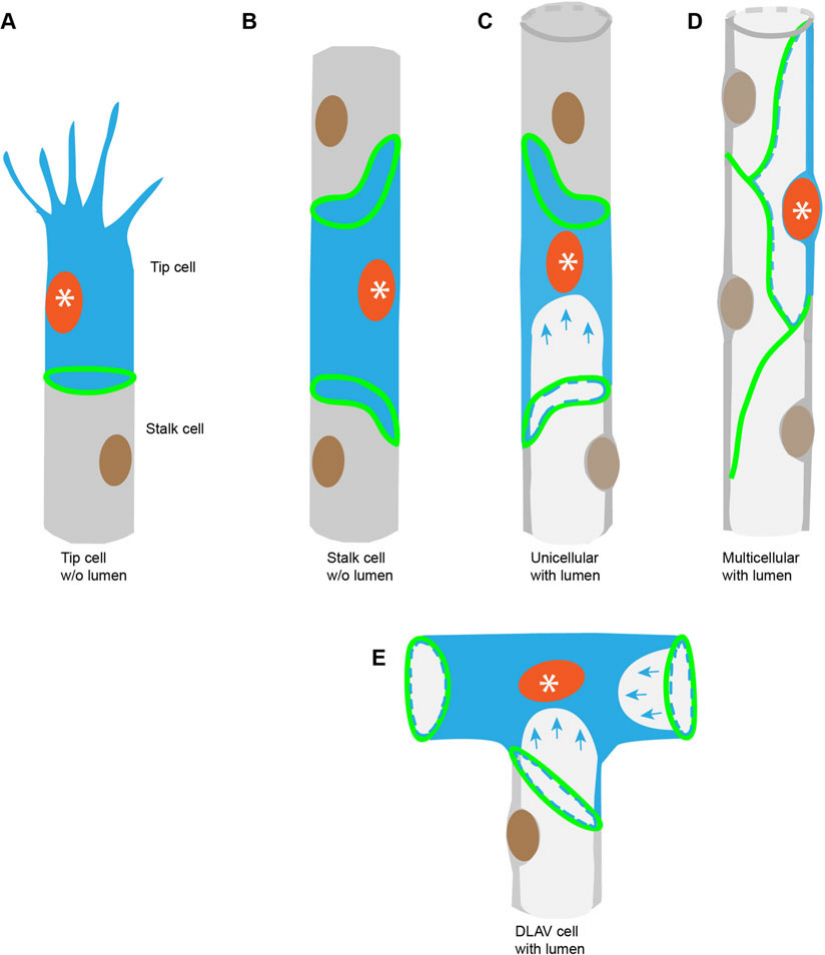
**Schematic representation of cell types in the SA and DLAV.** (A) Early sprout from the dorsal aorta, showing the shape of a tip cell, highlighted by blue cytoplasm and an orange nucleus. (B) Stalk cell with elliptical intercellular junctions, highlighted by blue cytoplasm and an orange nucleus, before lumen and DLAV formation. (C) Highlighted stalk cell (blue cytoplasm and orange nucleus) showing a partial transcellular lumen (blue arrows). (D) Fully lumenized multicellular SA, with one highlighted cell (blue cytoplasm and orange nucleus). (E) Cell within the DLAV shows the three-way connection with the neighboring cells and two partial transcellular lumens (blue arrows). (Green represents cell-cell junctions.)

The ECs that are part of the segmental arteries (SAs) divide in a stochastic fashion. SAs are dynamic structures, which contain anywhere between three and seven cells (Blum et al., 2008). Therefore, ECs, which undergo mitosis, may find themselves in quite different environments (Fig. 1). Tip cells sprout from the dorsal aorta (Childs et al., 2002; Siekmann and Lawson, 2007) and extend filopodia that will eventually contact those from neighboring branches, leading to anastomosis and giving rise to the DLAV (Blum et al., 2008; Herwig et al., 2011). During early phases of SAs formation the angiogenic sprout is not lumenized. Here, the tip cell is connected to the stalk cells by intercellular junctions (Fig. 1A). While the stalk is not lumenized at the onset of SAs sprouting, the stalk cells are surrounded by several neighbors – to each other, the tip cells and ventrally to ECs in the dorsal aorta (Fig. 1B). The formation of the lumen (through perfusion from the dorsal aorta) alters the shape of the stalk cells, as well as the relative positions of their nuclei and intercellular junctions (Fig. 1C). Subsequent junctional re-arrangements achieve the transition from a unicellular to a multicellular tube (Fig. 1D; Herwig et al., 2011; Lenard et al., 2013). Finally, the DLAV contains former tip cells that now possess a lumen and a T-shaped structure, and form intercellular junctions with the two neighboring tip cells and a stalk cell (Fig. 1E).

### Cell division in migrating tip cells

In order to document different aspects of EC proliferation in the ISVs, we imaged 30-50 hpf embryos of the following genotypes: (1) Tg(*fli1ep*:GAL4FF)^UBS3^, (UAS:RFP), (UAS:EGFP-ZO-1)^UBS5^; (2) Tg(*fli1ep*:GAL4FF)^UBS2-4^, (UAS:mRFP), (UAS:VE-cadherinΔC-EGFP)^ubs12^ (Herwig et al., 2011; Lenard et al., 2013); and (3) Tg(fli1ep:GAL4FF)^ubs3^; (UAS:mRFP); Tg(UAS:EGFP-UCHD)^ubs18^ (Sauteur et al., 2014). The EGFP-labeled ZO-1 (zona occludens 1) and VE-cadherinΔC-EGFP proteins localize to the cell junctions, EGFP-tagged UCHD (utrophin calponin homology domain) localizes to the stable F-actin, while cytoplasmic RFP labels the entire cell. The presence and extent of the lumen was ascertained by subtracting the volume occupied by the cytoplasm in the Z-axis, or through the localization of the membrane marker mKate2-CAAX (Lenard et al., 2013). Condensed chromatin was identified either as a zone of low RFP accumulation, or directly through the localization of labeled histones (H2B-GFP; Kochhan et al., 2013).

**Fig. 2.**
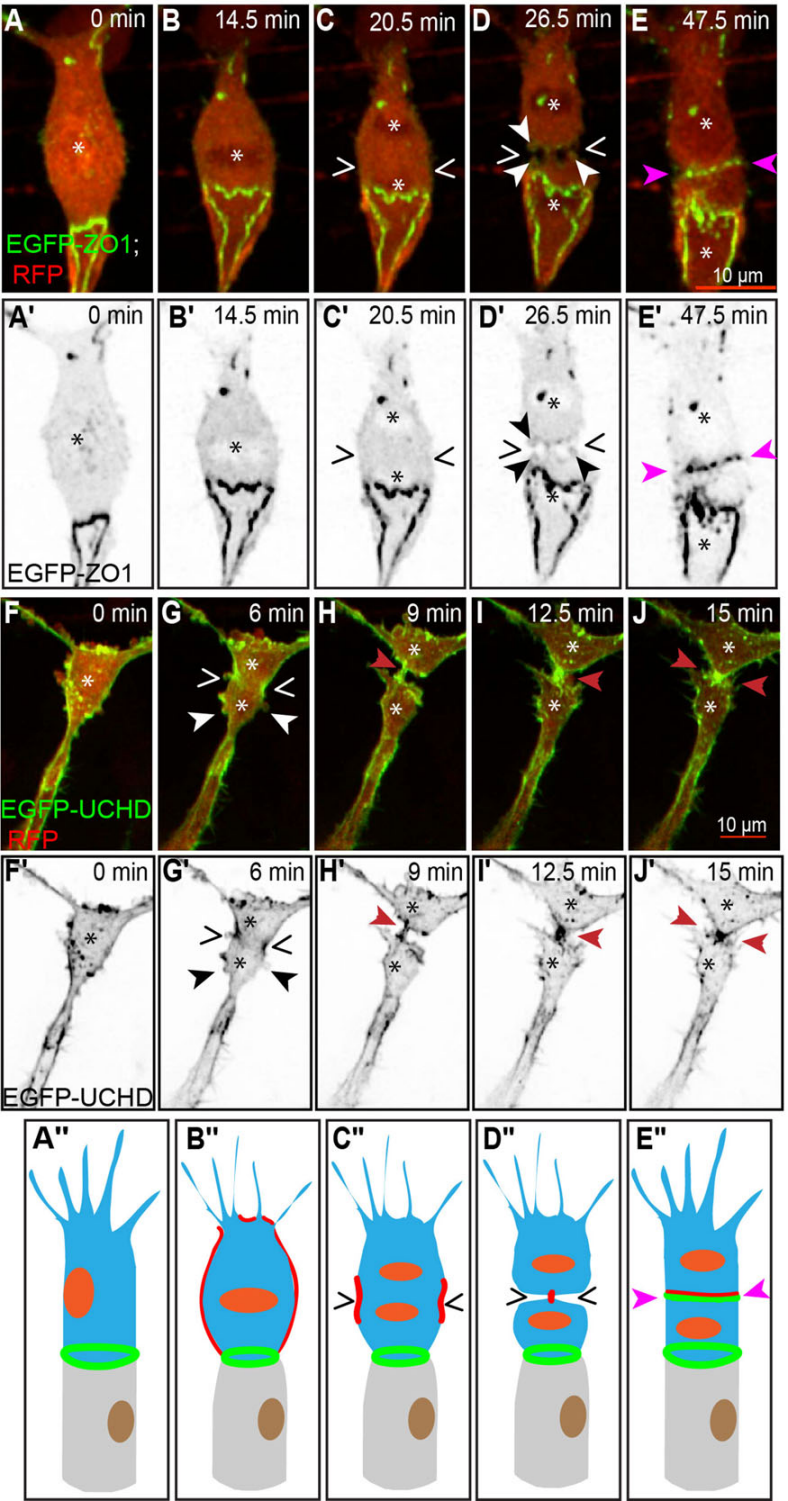
**Mitosis in a tip cell.** (A-E) Still pictures from time-lapse supplementary material Movie S1a, with highlighted intercellular junctions (EGFP- ZO1) and cytoplasm (RFP); (F-J) time-lapse supplementary material Movie S1b, with actin cytoskeleton (EGFP-UCHD) and cytoplasm (RFP). The location of condensed chromatin is apparent as a zone of decreased red signal (asterisks). (A) Partially rounded tip cell, with the approximate position of the nucleus indicated by the asterisk. (B) Fully rounded tip cell with a metaphase plate (asterisk). (C) Tip cell during anaphase, showing the separation of the daughter chromosomes (asterisks) and the beginning of membrane furrowing (wedges). (D) Late stage cytokinesis, with a narrow intercellular bridge in the plane of division (wedges). Blebs are visible at the interface between the daughter cells (white/black arrowheads). (E) Established *de novo* junction between the two daughter cells (pink arrowheads). (A′-E′) Still pictures corresponding to those from panels A-E, showing only the green channel (grayscale). (F) Partially rounded tip cell, with the approximate position of the nucleus indicated by the asterisk. (G) Tip cell during anaphase, showing the separation of the daughter chromosomes (asterisks) and the beginning of membrane furrowing with actin accumulation (wedges) and blebs are visible at the cell surface (white/black arrowheads). (H) Late stage cytokinesis, with a narrow connection bridge in the plane of division (red arrowheads). (I) Thickening of the connection bridge between the daughter cells (red arrowheads). (J) Connection formed between the two daughters cells (red arrowheads). (F′-J′) Still pictures corresponding to those from panels F-J, showing only the green channel (grayscale). (A″-E″) Schematic representation corresponding to the five stages from panels A-E. (B″-E″) Schematic representation corresponding to from panels F-J. (In the schematic: red, actin; green, junctions; orange, nucleus; blue, cell body.)

During the early stages of SA formation, tip cells extend filopodia structures can undergo rapid shape changes. We observed that dividing tip cells (*n*=15; where *n* represents the number of the examined vessels) underwent mitotic rounding (Fig. 2B,B′,F,F′). At anaphase, cell furrowing started symmetrically (Fig. 2C,C′,G,G′, wedges) from both sides and was accompanied by actin accumulation. Following anaphase (Fig. 2C,C′), the cell was pinched the cell body at the plane of division (Fig. 2D,D′; supplementary material Movie S1a) and the two daughter cells remained attached through a thin cytoplasmic bridge (Fig. 2H,H′; supplementary material Movie S1b). We observed the deposition of new junctional material in the region of the division plane, followed by its expansion as the two daughter cells established a *de novo* junction along the mitotic interface. Blebs (Fig. 2D,G, arrowheads), which are dynamic membrane protrusions generated through actomyosin contractions (Bergert et al., 2012), were present at the interface between the daughter cells during cytokinesis. Interestingly, the filopodial dynamics at the front of the tip cells was not markedly changed during cell division, despite the mitotic rounding of the cell body (supplementary material Movie S1c).

### *De novo* junctional formation in dividing stalk cells without a lumen

**Fig. 3.**
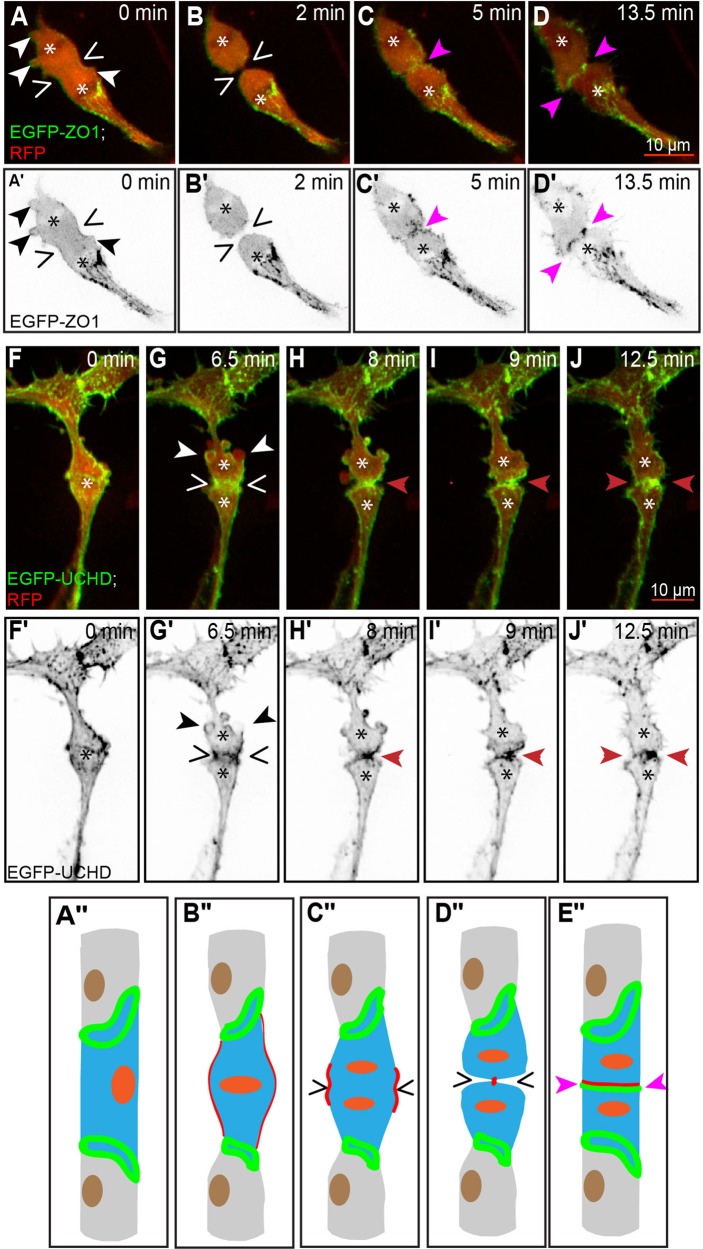
**Mitosis in a stalk cell without a lumen.** (A-E) Still pictures from time-lapse supplementary material Movie S2a, with highlighted intercellular junctions (EGFP- ZO1) and cytoplasm (RFP); (F-J) time-lapse supplementary material Movie S2b, with actin cytoskeleton (EGFP-UCHD) and cytoplasm (RFP). The location of condensed chromatin is apparent as a zone of decreased red signal (asterisks). (A) Stalk cell during anaphase, showing the separation of the daughter chromosomes (asterisks) and the beginning of membrane furrowing (wedges). Blebs are visible at several locations around the cell (white/black arrowheads). (B) Late stage cytokinesis, with a narrow intercellular bridge in the plane of division (wedges). (C) Established *de novo* junction between the two daughter cells (pink arrowheads), as the two daughter cells start to reconnect. (D) Wide intercellular junction between the two daughter cells. (A′-D′) Still pictures corresponding to those from panels A-D, showing only the green channel (grayscale). (F) Partially rounded stalk cell, with the approximate position of the nucleus indicated by the asterisk. (G) Stalk cell during anaphase, showing the separation of the daughter chromosomes (asterisks) followed by blebs formation (white/black arrowheads) and the beginning of membrane furrowing with actin accumulation (wedges). (H) Late stage cytokinesis, with a narrow connection bridge in the plane of division (red arrowheads). (I) Thickening of the connection bridge between the daughter cells. (J) Connection formed between the two daughters cells (red arrowheads). (F′-J′) Still pictures corresponding to those from panels F-J, showing only the green channel (grayscale). (A″,B″) Schematic representation of the stalk cell during prophase and metaphase, not part of the movie (A-D). (C″-E″) Schematic representation corresponding to panels A-D. (A″-E″) Schematic representation corresponding to panels F-J. (In the schematic; red: actin, green: junctions, orange: nucleus, blue: cell body).

We next investigated those stalk cells, which divided before acquiring a lumen. We observed that dividing stalk cells (*n*=10; Fig. 3F,F′) also underwent mitotic cell rounding. Although lacking the extended filopodia of tip cells, these stalk cells also produced numerous blebs during mitosis (Fig. 3A,G, white/black arrowheads). Cytokinesis took place symmetrically and perpendicularly to the long axis of the sprout (Fig. 3A,G). Subsequently, and similar to tip cells, the two daughter cells formed a *de novo* junction (Fig. 3C,C′,D,D′; supplementary material Movie S2a) and actin accumulated at the plane of division (Fig. 3I,I′,J,J′; supplementary material Movie S2b).

### Dividing unicellular stalk cells collapse the lumen in the plane of mitosis

**Fig. 4.**
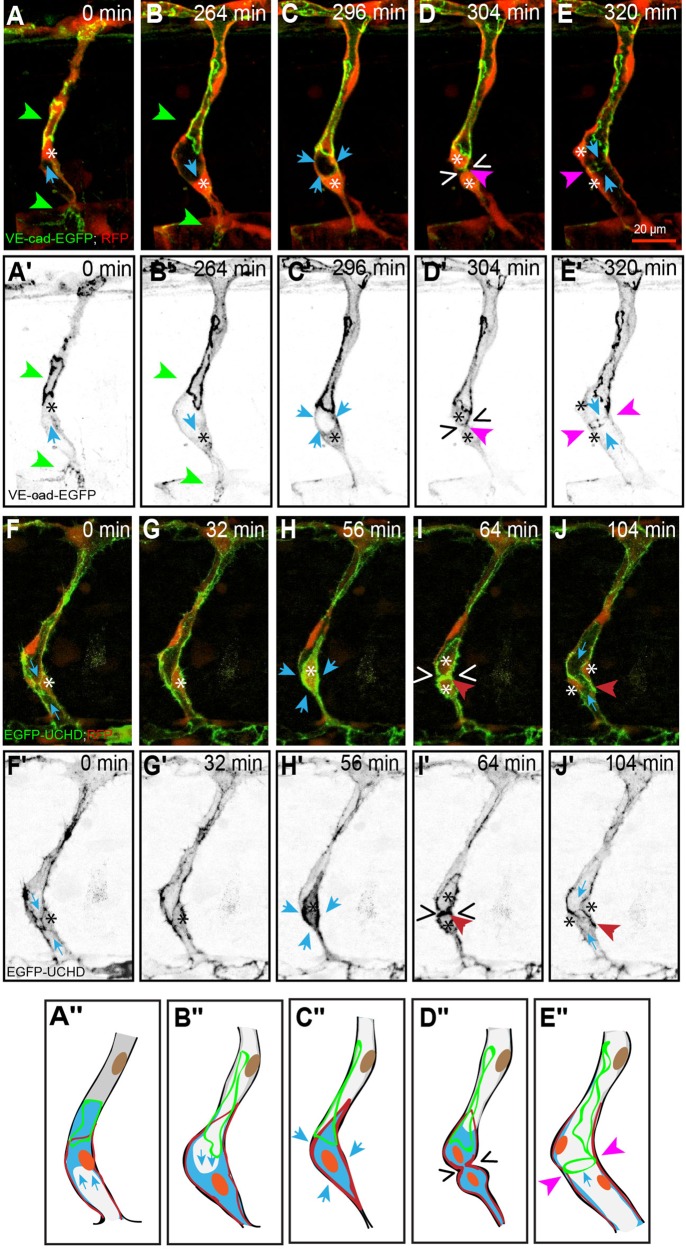
**Mitosis in a stalk cell with a partial transcellular lumen.** (A-E) Still pictures from time-lapse supplementary material Movie S3a, with highlighted intercellular junctions (VE-cadherinΔC-GFP) and cytoplasm (RFP); (F-J) time-lapse supplementary material Movie S3b, with actin cytoskeleton (EGFP-UCHD) and cytoplasm (RFP). The location of the lumen is apparent as an empty zone bounded by red cytoplasm (blue arrows), and the approximate position of the nuclei is indicated by asterisks. (A) Stalk cell with a partial transcellular lumen (blue arrow) that extends from the aorta to the nucleus (asterisk). The cell is bounded by ring-like intercellular junctions (green arrowheads). (B) Retraction of the lumen from the bottom side of the cell and expansion of the transcellular lumen from the top (blue arrow). Elongation of the top ring and contraction of the bottom ring (green arrowheads). (C) Rounding of the stalk cell pushes out the lumen from the nuclear region (blue arrows). (D) Late stage cytokinesis, with a narrow intercellular bridge in the plane of division (wedges). The *de novo* junction between the two daughter cells is apparent as a dot (pink arrowheads) close to the intercellular bridge that contains the midbody. (E) The lumen re-inflates after mitosis is complete (blue arrows), expanding the *de novo* junction between the two daughter cells into a ring (pink arrowheads). (A′-E′) Still pictures corresponding to those from panels A-E, showing only the GFP channel (grayscale). (F) Stalk cell with a transcellular lumen (blue arrows) that extends from the aorta to the nucleus (asterisk). The cell is bounded by actin cytoskeleton. (G) Narrowing of the lumen from the bottom side of the cell prior to division. (H) Rounding of the stalk cell collapses the lumen from the bottom side of the cell (blue arrows). (I) After cytokinesis (wedges), the actin cytoskeleton accumulated at the plane of division (red arrowhead). (J) The lumen re-inflates after mitosis is complete (blue arrows), expanding the actin cytoskeleton between the two daughter cells (red arrowheads). (F′-J′) Still pictures corresponding to those from panels F-J, showing only the green channel (grayscale). (A″-E″) Schematic representation corresponding to the five stages from panels A-E and F-J. (In the schematic: red, actin; green, junctions; orange, nucleus; blue, cell body.)

We also investigated unicellular tubes made up of stalk cells that contain a lumen and entered mitosis (*n*=10). Such unicellular stalk cells form a transcellular lumen through a process of apical membrane invagination, during which the luminal compartment is extended and inflated from one or both sides of the cell under the pressure of incoming blood plasma. This type of lumen is not very stable and can easily collapse, upon a local drop in pressure (Herwig et al., 2011; Lenard et al., 2013). We observed that – in many cases – the inflated lumen did not penetrate the region of the cell where the prophase nucleus was located (Fig. 4A′; *n*=10). In those instances, in which comprised regions on either side of the nucleus, and where a pressure differential developed between the two sides, the nucleus was in some cases shifted to a new location within the cell (Fig. 4A,B; supplementary material Movie S3a). We also followed cell division in the presence of a fully inflated transcellular lumen (Fig. 4F,G; supplementary material Movies S3b, S8). The section of the lumen adjacent to the nucleus shrank during mitotic rounding (Fig. 4C,C′,H,H′). Subsequently, the lumen was entirely excluded from that region for the duration of cytokinesis (Fig. 4D,D′, I,I′), only to return immediately after the completion of mitosis (Fig. 4J,J′) and thereby inflating the ring-like *de novo* junction (Fig. 4E,E′,J,J′).

### Mitosis in multicellular SAs does not collapse the lumen

**Fig. 5.**
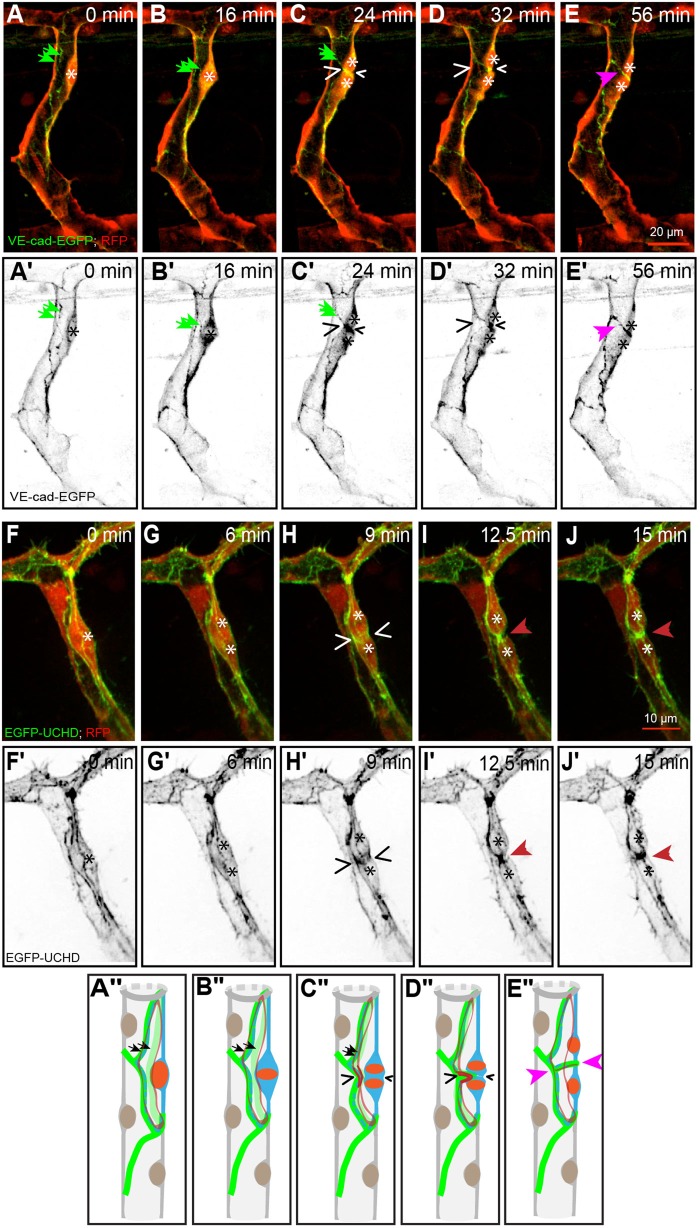
**Mitosis in a stalk cell with an extracellular lumen.** (A-E) Still pictures from time-lapse supplementary material Movie S4a, with highlighted intercellular junctions (VE-cadherinΔC-GFP) and cytoplasm (RFP); (F-J) time-lapse supplementary material Movie S4b, with actin cytoskeleton (EGFP-UCHD) and cytoplasm (RFP). The location of the lumen is apparent as an empty zone bounded by red cytoplasm, and asterisks indicate the approximate position of the nuclei. (A) Stalk cell with intercellular junctions surround whole surface of a cell body (green arrows) that is part of a multicellular tube, prior to mitosis. (B) Stalk cell during metaphase, showing that the intercellular junctions (green arrows) and the extracellular lumen are not affected by the mitotic rounding. (C) Following anaphase, the furrowing of the actomyosin ring (wedges) symmetrically deforms the adjacent intercellular junctions (green arrows). (D) Late stage cytokinesis, with a narrow intercellular bridge in the plane of division (wedges). The angle between the deformed intercellular junctions has become more acute. (E) Formation of a new junction between the daughter cells (pink arrowheads), through the modification of the existing intercellular junctions. (A′-E′) Still pictures correspond to those from panels A-E, showing only the green channel (grayscale). (F) Partially rounded stalk cell that is a part of multicellular tube, prior to mitosis and the extracellular lumen are not affected by the mitotic rounding. (G) Stalk cell during anaphase, showing the separation of the daughter chromosomes (asterisks). (H) The beginning of membrane furrowing with actin accumulation (wedges). (I) Late stage cytokinesis, with a narrow connection bridge in the plane of division (red arrowheads). (J) Thickening of connection bridge between the daughter cells and connection formed between the two daughters cells (red arrowhead). (F′-J′) Still pictures corresponding to those from panels F-J, showing only the green channel (grayscale). (A″-E″) Schematic representation corresponding to the five stages from panels A-E and F-J. (In the schematic: red, actin; green, junctions; orange, nucleus; blue, cell body; black arrows, position of junctions.)

During the later stages of SA formation, when the DLAV has formed and blood flow has been established, most ECs are part of multicellular tubes. In these vessels, we observed that dividing stalk cells (Fig. 5A,A′) clearly undergo mitotic rounding as well. However, and in contrast to cell division in a unicellular, lumenized tube, the lumen was kept intact during the entire process of cytokinesis (Fig. 5B,B′, *n*=25). The behavior of the intercellular junctions in such tubes closely resembled those described in epithelial tissues (Bourdages and Maddox, 2013). We observed that during anaphase, actin accumulated in the division plane, at a spot where two adjacent junctions were pulled towards the prospective location of the midbody, halfway between the two daughter nuclei (Fig. 5C,C′,H,H′). The previously straight junctions of the dividing cell were bent and brought into close proximity during cytokinesis (Fig. 5D,D′,I,I′; supplementary material Movie S4a,b), and shortly thereafter, the new intercellular junction began to form. Therefore, the new junctions only needed to extend halfway around the lumen in multicellular tubes (Fig. 5E,E′). Cytokinesis took place symmetrically, as the division plane that contained the collapsing actomyosin ring was equally distant to the two sides of the cell.

### ECs within the DLAV can undergo asymmetric cytokinesis

**Fig. 6.**
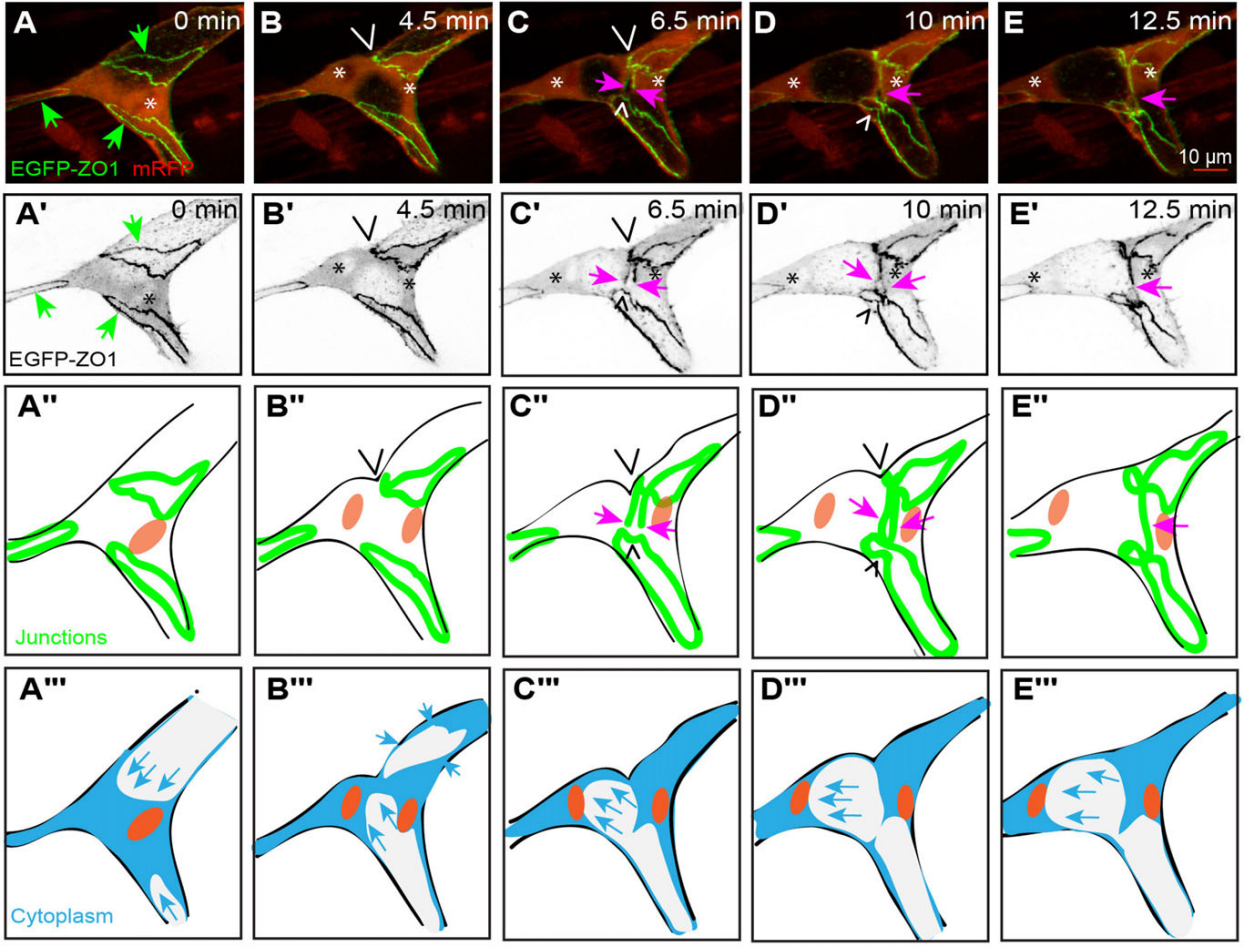
**Mitosis in a cell within the DLAV with a partial transcellular lumen.** (A-E) Still pictures from time-lapse supplementary material Movie S5, with highlighted intercellular junctions (EGFP-ZO1) and cytoplasm (RFP). The location of the lumen is apparent as an empty zone bounded by red cytoplasm, and the approximate position of the nuclei is indicated by asterisks. (A) T-shaped cell within the DLAV with intercellular junctions (green arrows), during the metaphase. (B) Lumenal push, from the bottom, positions the nucleus close to the top ring and following anaphase furrowing is observed in the upper region of the cell (wedge). (C) The top ring (wedges) deforms during the anaphase, generating a pair of cellular processes that extend towards the opposite side of the cell (pink arrows). The lumen is partially constricted in the plane of division. (D) During late stage cytokinesis, the bottom ring is deformed as well (wedge) prior to the closure of the new junction (pink arrow). The lumen continues to shrink within the plane of division, without completely collapsing. (E) A complete, ring-like junction is established between the daughter cells (pink arrow), through the modification of the existing intercellular junctions. (A′-E′) Still pictures corresponding to those from panels A-E, showing only the green channel (grayscale). (A″-E″) Schematic representation corresponding to the five stages from panels A-E, depicting the nuclei and the junctions. (A‴-E‴) Schematic representation corresponding to the five stages from panels A-E, depicting the nuclei and the cytoplasm/lumen. (In the schematic; green: junctions, orange: nucleus, blue: cell body, blue arrows: ending and growth direction of lumen.)

During anastomosis, tip cells establish junctions with their anterior and posterior counterparts along the DLAV, while retaining their initial junction with the most distal stalk cell of the corresponding sprout giving rise to T-shaped ECs. These ECs have 3 junctional rings and underwent asymmetrical cytokinesis, depending on the location of the prophase nucleus relative to the junctional rings. The DLAV was shown to transition from a unicellular to a multicellular tube through junctional re-arrangement (Herwig et al., 2011). Before this transition occurs, cells within the DLAV may be partially or completely perfused by a lumen, which in turn may be connected to the SA stalk, the neighboring tip cells, or both (Fig. 6A-A‴). The expansion of lumen (derived from the SA) into the dividing cell was apparent during metaphase and anaphase (Fig. 6A‴,B‴). Subsequently, the progression of cytokinesis was associated with the shrinkage of the lumen in the plane of division (Fig. 6C,D,C‴,D‴). We observed the preferential deformation of the intercellular junction closest to the mitotic nucleus during the early stages of cytokinesis (Fig. 6B-B″). The emerging new junction first appeared as a pair of cellular processes, derived from the top right junctional ring (Fig. 6C-C″; supplementary material Movie S5). After the growing junction spanned almost the entire width of the cell, a complementary deformation of the bottom junctional ring became apparent as well (Fig. 6D-D″), shortly before the completion of the new junction between the daughter cells (Fig. 6E-E″). The cell within the DLAV divided without completely collapsing the lumen, in a manner reminiscent of the ECs in multicellular vessels. However, the repositioning of the nucleus close to one of the intercellular junctions favored asymmetrical cytokinesis. Our analysis (*n*=5) indicates that the actomyosin ring preferentially deforms those junctions that are close to it during cytokinesis (Fig. 6C-C″).

### New junctions form through the zipping of furrowed membranes and the lumen does not impede cytokinesis

**Fig. 7.**
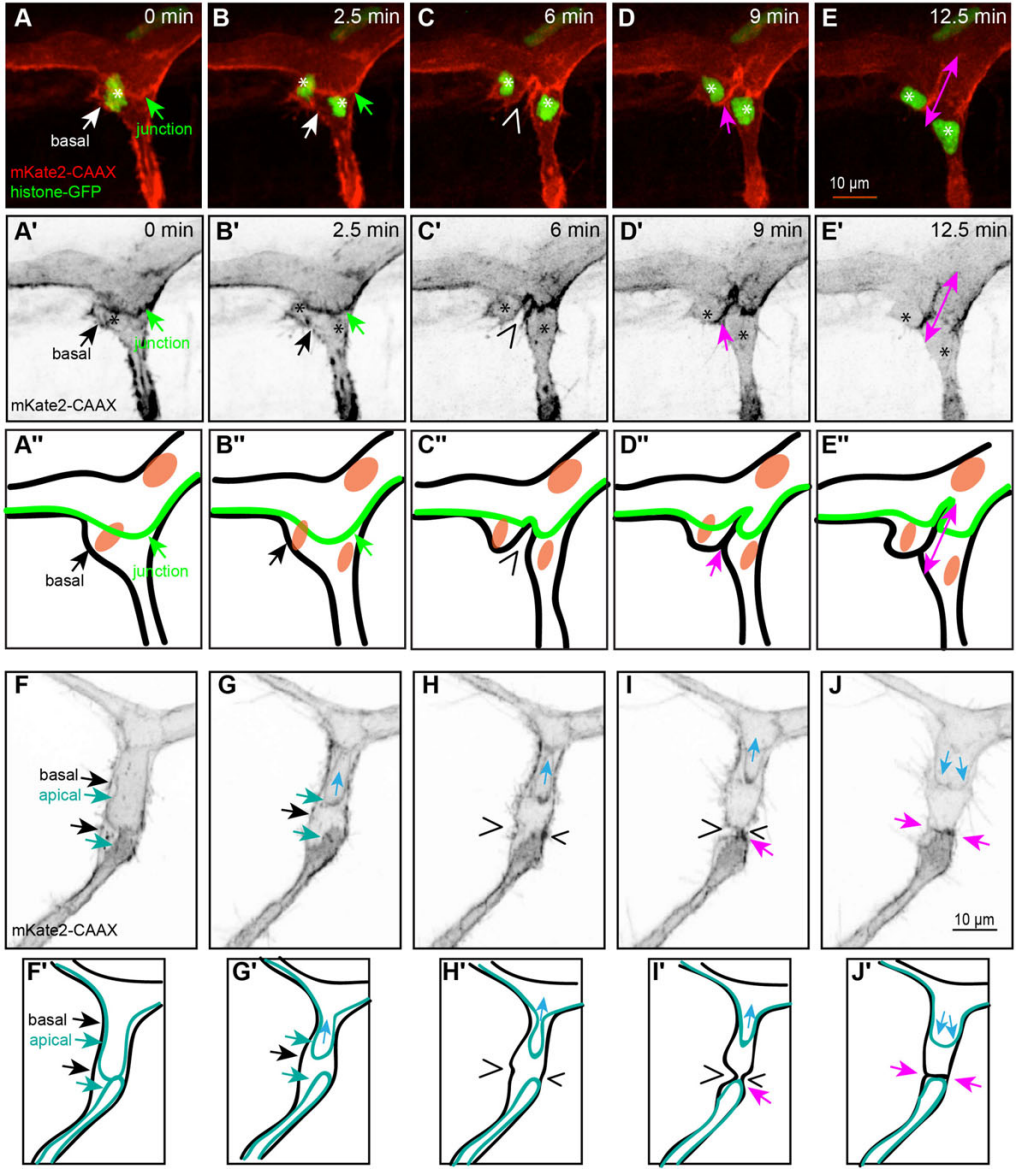
**Membrane furrowing and lumen dynamics in a dividing cell from the DLAV.** (A-E) Still pictures from time-lapse supplementary material Movie S6, with highlighted membranes (mKate2-CAAX). The apical side (where the lumen is located) is apparent as a bright zone bounded by cell membranes, cell-cell contacts form a stripe of strong red signal (green arrow) and the position of the nuclei is shown by histone-GFP and asterisks. (A) Cell within the DLAV with a nucleus on the left (asterisk represents nuclei number.) connected to the ISV cells (intercellular junction, green arrow), prior to mitosis. (B) Cell after anaphase and before the start of cytokinesis. The daughter nuclei have separated but the membranes have not furrowed. (C) The membrane starts furrowing (wedge) asymmetrically during cytokinesis, generating a deep furrow that extends towards the middle of the vessel. (D) During late stage cytokinesis, the two sides of the membrane are juxtaposed (pink arrow). (E) The new junction between the two daughter cells forms through the zipping of the juxtaposed membrane, starting from the apical side (pink double arrow). (A′-E′) Still pictures corresponding to those from panels A-E, showing only the red channel (grayscale). (A″-E″) Schematic representation corresponding to the five stages from panels A-E, depicting cell-cell contacts (green line and arrow), the basal membrane (black) and the nuclei (orange). (F-J) Still pictures from time-lapse supplementary material Movie S7, with highlighted membranes (mKate2-CAAX). The apical side (where the lumen is located) is apparent as an intracellular membrane (turquoise arrows). (F) Cell within the DLAV, showing the position of the apical (turquoise arrows) and basal (black arrows) membranes, prior to mitosis. The region contains an upper trans-cellular lumen and a lower multicellular lumen. (G) The cell undergoes mitotic rounding and the upper transcellular lumen is pushed away from the future plane of mitosis (light blue arrow), while the lower multicellular lumen constricts but is not excluded from the region. (H) The cell after anaphase and before the start of cytokinesis. The upper lumen continues to shrink (light blue arrow) while the multicellular lumen remains unaffected. (I) The membrane starts furrowing (black wedges) asymmetrically deforms the basal membrane, with a deeper furrow on the left side of the cell, which is more distant from the multicellular junction on the right side. (J) Formation of a *de novo* junction between the two daughters cells (pink arrows). Following the end of mitosis, the upper lumen recovers and begins to push again into the vessel. (F′-J′) Schematic representation corresponding to the five stages from panels F-J, depicting the apical membrane (turquoise lines and arrows), the basal membrane (black/white arrows represent basal membrane).

In order to better understand how the new junctions are formed during asymmetrical cytokinesis, we investigated the dynamics of the cell membrane on the apical and basolateral side, using the Tg(BAC:*kdrl*:mKate2-CAAX)^UBS16^ marker (Lenard et al., 2013) together with the Tg(*kdrl:H2B-GFP*) (Kochhan et al., 2013) marker, which label cell membranes and chromatin, respectively. The mitotic rounding of the dividing cell did not deform the lumen inflated between the two cells (Fig. 7A-A″). According to our observations, the membrane furrowing that is presumably driven by the contraction of the actomyosin ring begins on the basolateral (outer) side of the vessel and proceeds towards its middle, where the cell connects to another EC, thereby supporting the lumen (Fig. 7C-C″; supplementary material Movie S6). During the late stages of cytokinesis, when the two sides of the membrane have been juxtaposed (Fig. 7D-D″), the new junction zips together (Fig. 7E-E″; supplementary material Movie S6).

Using the same membrane marker, we followed the lumen dynamics during cytokinesis in a cell that possesses a transcellular lumen, in addition to a dead-end multicellular lumen that is supported together with a neighboring cell (Fig. 7F). As previously described in the article, the apical side that faces the lumen is visible as a nested membrane within the tube, while the basolateral membrane is seen on the outside of the blood vessel. The two lumens were initially in close proximity. However, at the onset of mitotic rounding of the transcellular lumen compartment collapsed, while the proximal multicellular luminal compartment appeared unchanged, apart from a degree of narrowing (Fig. 7G,G′). We observed that membrane furrowing was asymmetrical, on account of the position of the lower neighboring cell, relative to the plane of division (Fig. 7I,I′; supplementary material Movie S7). Following the establishment of a *de novo* junction and the completion of mitosis, both lumens began to expand, especially the upper transcellular lumen (Fig. 7J,J′; supplementary material Movie S7). Therefore, the transcellular lumen present within the unicellular tubes was found to be responsive to the changes in cell shape accompanying the mitotic process, and did not interfere with cytokinesis.

## DISCUSSION

To date, the largest body of *in vivo* data on mitosis in a multicellular environment has been collected in *Drosophila* and describes the process in the embryonic epithelium (Guillot and Lecuit, 2013), the wing disc epithelium (Nakajima et al., 2013), the follicular epithelium (Morais-de-Sá and Sunkel, 2013), the dorsal thorax epithelium (Founounou et al., 2013; Herszterg et al., 2013) and the trachea (Denes et al., 2015; Kondo and Hayashi, 2013). With our analysis of cell division in the vasculature of the zebrafish embryo, we aimed to characterize those aspects of the mitotic process that are conserved between the tubular organs of vertebrates and those of insects. We have identified a sequence of cellular events that appears to be shared between the two cellular systems, which is not surprising given that all epithelial cells face a similar set of constraints, such as retaining their apico-basal polarity and their barrier function throughout mitosis. In our study, we have examined the specific interactions between the lumen, the junctions and the actin distribution, within the different cellular architectures that are encountered in the embryonic zebrafish trunk vasculature (Fig. 1).

The sprouting of the tip cells from the dorsal aorta is the first step towards the formation of the SAs and the DLAV. Branch elongation takes place through a combination of tip cell migration and actin-mediated shape changes within the stalk cells (Sauteur et al., 2014). In most cases, the nascent sprout is not lumenized at this early stage (*n*=25). At this stage, ECs may undergo mitosis with little interference from the neighboring cells, as their intercellular junctions are located toward the proximal and distal tips of the cells, and therefore away from the future mitotic plane (Fig. 2A and Fig. 3A). These dividing cells were observed to go through the ‘classic’ stages, namely mitotic rounding during metaphase (Cadart et al., 2014), symmetrical membrane furrowing during cytokinesis (Green et al., 2012), and expansion of cell-cell contacts as the first step of *de novo* junction formation (Yamada and Nelson, 2007).

Whereas the formation of multicellular tubes depends on stalk cell elongation, which allows extensive ‘cell pairing’, in the absence of this pairing a unicellular tube can be formed via transcellular lumen formation. The mechanism of transcellular lumen formation depends by and large on directed membrane invagination along the blood vessel axis. Because of the small diameter of ECs, endothelial nuclei may impede this invagination process (Fig. 4A-A″). Despite the plasticity of these ECs, the lumen cannot easily penetrate through the region containing the nucleus, and under certain conditions, the nucleus may also be shifted back and forth within the cell, similar to a piston in a cylinder (Fig. 4A-B). During metaphase, the lumen is eventually excluded from the plane of division. However, the lumen is capable of re-perfusing the two daughter cells immediately after mitosis is complete (Fig. 4E). These dynamic changes of luminal compartments are consistent with our previous reports. Lumen forms and collapses repeatedly in blood vessels with erratic blood flow, during the early steps of vessel fusion (Lenard et al., 2013) or during vessel regression (Lenard et al., 2015). Cell division causes changes in the organization of the cytoskeleton, which may account for the instability of the generally fragile transcellular lumen.

The simplest multicellular tubes are composed of a pair of cells that envelop a lumen and are held together by continuous intercellular junctions along the vessel axis. Unlike the partial transcellular lumens, the extracellular lumen of multicellular tubes is more robust and does not collapse during mitotic rounding. We have also observed that the plane of division was always orthogonal to the axis of the lumen, thereby positioning the actomyosin ring close to the intercellular junctions. Within planar epithelial sheets, the neighboring cells that are flanking the mitotic plane and the associated actomyosin ring exert a strong influence on the progression of cytokinesis. The actin cytoskeleton of the neighboring cells is coupled to the plasma membrane through cadherin proteins, which are in turn the core components of adherens junctions (Harris and Tepass, 2010). In order for cytokinesis to take place, the ingression of the actomyosin ring must overcome the tensile forces generated by the neighboring cells (Bourdages and Maddox, 2013). However, it has also been shown that the juxtaposition of the furrowed membrane is actively supported by the neighboring cells, through actin polymerization at the base of the furrow (Founounou et al., 2013; Herszterg et al., 2013). Our analysis of cytokinesis in multicellular tubes reveals that membrane ingression takes place symmetrically on both sides of the contracting actomyosin ring (Fig. 5C-C″). The membrane of the counterpart cell undergoes a complementary deformation, ensuring that adhesion and barrier function are maintained for the duration of mitosis. We were able to investigate the localization of actin filaments and junctions in different cells. The sharp shoulders of the furrowed membrane (Fig. 5D′) are very similar to those described in planar epithelial sheets (Herszterg et al., 2014), which suggest that cytokinesis in tubular epithelia is probably a multicellular process as well.

Cells within the DLAV have a more balanced aspect ratio compared to stalk cells, with a central body and 3 sets of ring-shaped intercellular junctions at the tips of a T shape. Lumen perfusion can occur from either direction and on account of their three-fold symmetry, the prophase nucleus may be positioned close to only one of the rings (Fig. 6A,B). In such an orientation, the actin accumulates preferentially at an asymmetric position and the actomyosin ring preferentially deforms the intercellular junction that was adjacent or closest to the plane of division, resulting in an asymmetric cytokinesis event (Fig. 6C). Tubular cells from the trachea of *Drosophila* larvae have recently been shown to undergo such asymmetric cytokinesis (Denes et al., 2015). In both instances, the ingression of the actomyosin ring caused a local deformation of the junction, followed by the formation of a membrane protrusion that grew until it contacted another junction, where they finally connected (Fig. 6D). However, it is important to note that the rigid lumen found in the trachea forces the emerging junction to curve around it (Denes et al., 2015), whereas in the DLAV, the lumen slowly yields to the tension generated by cytokinesis (Fig. 6E,E‴).

Mitotic cells from polarized epithelia have also been shown to undergo asymmetric cytokinesis along the apico-basal axis. Because the cytokinetic furrow contracts faster from the basal side, the midbody eventually comes to rest at the apical side of the dividing cell (Founounou et al., 2013; Guillot and Lecuit, 2013). Our analysis shows that membrane ingression in cells within the DLAV takes place asymmetrically (Fig. 7C) and that the zipping of the two juxtaposed membranes begins at the apical side, where the lumen is located (Fig. 7D,E).

### Conclusion

We found that in the absence of a lumen, endothelial cells in the segmental arteries divide symmetrically and form intercellular junctions *de novo*. The mechanisms of cell division in multicellular tubes closely resemble those seen in planar epithelial sheets: following actomyosin ring ingression and membrane furrowing, new junctions between the daughter cells are formed through the modification of pre-existing ones. However, we also observed asymmetric cytokinesis in T-shaped cells within the DLAV, in which the prophase nuclei were positioned closer to some intercellular junctions than to others.

## MATERIALS AND METHODS

### Zebrafish maintenance and strains

Zebrafish (*Danio rerio*) were maintained at standard conditions (Westerfield, 2007) and embryos were staged at 28.5°C, as previously described (Kimmel et al., 1995). The following transgenic lines were used in this study: Tg(fli1ep:gal4ff)^ubs3^ Tg(UAS:EGFP-ZO1-cmlc:EGFP)^UBS5^ (Herwig et al., 2011), Tg(5×UAS:mRFP) (Asakawa et al., 2008), Tg(BAC:*kdrl*:mKate2-CAAX)^UBS16^ and Tg(UAS:VE-cadherinΔC-EGFP)^ubs12^ (Lenard et al., 2013), Tg(*kdrl:H2B-GFP*)^mu122^ (Kochhan et al., 2013), Tg(UAS:EGFP-UCHD)^ubs18^ (Sauteur et al., 2014).

### *In vivo* time-lapse analysis

Staged embryos that exhibited fluorescence were anaesthetized with tricaine solution (0.08%) and subsequently mounted in a 35-mm glass bottomed petri dish (0.17 mm, MatTek), containing 0.7% low-melting agarose (Sigma), 0.08% tricaine and 0.003% PTU (Sigma). Long duration movies were recorded with a Leica TCS SP5 confocal microscope, using a 40× (NA=1.1) water immersion objective. Z-stacks with a 0.7- to 1-µm step size were acquired every 8 or 10 min.

High-resolution movies were acquired with a spinning disk confocal microscope (Perkin Elmer Ultraview), using a 63× (NA=1.2) water immersion objective. Z-stacks with a 0.2-µm step size were recorded every 30 s. For improved resolution, datasets were deconvolved using the Huygens Remote Manager software (Ponti et al., 2007).

Imaris (Bitplane), Volocity (PerkinElmer) and ImageJ (http://imagej.nih.gov/ij/) were used for additional data processing and analysis. All images are maximum intensity projections.
